# Voluntary wheel-running exercise improvement of anxiety or depressive symptoms in different models of depression

**DOI:** 10.3389/fnbeh.2024.1435891

**Published:** 2024-10-15

**Authors:** Haodi Shen, Xuemei Li, Junyao Zhai, Xin Zhang

**Affiliations:** College of Sports Medicine and Rehabilitation, Beijing Sport University, Beijing, China

**Keywords:** voluntary wheel-running exercise, depression models, forced swimming test, sucrose preference test, elevated plus maze

## Abstract

The effects of voluntary wheel-running exercise in different rodent models of depression remain unclear, and further research is needed to fully understand the mechanisms underlying these effects. Therefore, this systematic review aimed to evaluate the currently available findings on whether voluntary wheel-running exercise can alleviate depressive symptoms in five different rodent models of depression. The findings of the comprehensive meta-analysis imply that engaging in voluntary wheel-running exercise has a beneficial effect on alleviating depressive symptoms in rodent models that simulate depression. While further research is needed to fully understand the mechanisms and limitations of this intervention future research should aim to conduct larger. Well-designed studies that use standardized protocols and outcome measures. This would help to reduce heterogeneity between studies and improve the overall quality of the evidence base. Additionally, studies should explore the potential mechanisms of action of voluntary wheel-running exercise in treating depression, such as changes in neurotransmitter levels, neuroplasticity, and inflammation. The results suggest that it may hold promise as an adjunctive therapy for depression.

## 1 Introduction

The increasing prevalence of depression worldwide, with predictions of it becoming one of the top three burdensome diseases by 2030, underscores the urgent need for effective treatment options. The high recurrence rate of depression and its significant economic burden on individuals and society highlight the importance of finding new therapeutic approaches (Malhi and Mann, 2018; Mathers and Loncar, 2006; Zhdanava et al., 2021). Voluntary wheel-running exercise has emerged as a promising non-pharmacological intervention in animal models of depression. Its rewarding and voluntary nature, along with its ability to mimic natural running patterns and minimal stress on animals, make it an attractive option for long-term studies.

Several modeling methods exist to study depression, each simulating different stressors and scenarios that can lead to depressive symptoms. The Maternal Separation model focuses on early life trauma (Fellmeth et al., 2018; Liu et al., 2023; Ding et al., 2019), the CUMS model simulates work-related stress and sedentary lifestyles (Alreshidi and Rayani, 2023; Marzouk et al., 2018; Baglioni et al., 2016; Hoare et al., 2016; Lee and Kim, 2019), and the Social Defeat model mimics bullying and domestic violence (Husky et al., 2020; Azúa Fuentes et al., 2020). These models offer valuable insights into the complex nature of depression and its various triggers (Wu et al., 2020; Naghshvarian et al., 2017; Mul, 2018; Harro, 2019).

However, it remains unclear whether voluntary wheel-running exercise has a universal therapeutic effect across these different depression models. This meta-analysis aims to bridge this knowledge gap by synthesizing existing evidence on the potential benefits of voluntary wheel-running exercise for different depression models. By doing so, we hope to provide a more comprehensive understanding of the role of exercise in depression treatment and inform future research and clinical practice.

## 2 Materials and methods

### 2.1 Search and strategy

We searched English databases, such as PubMed, Web of Science, Embase, and EBSCOhost, to find relevant articles. The search was conducted from the establishment of the database until April 16, 2024. We used a combination of subject terms and free words tailored to each database's characteristics in our search. The English keywords we used were (“voluntaries”[All Fields] OR “voluntary”[All Fields]) AND (“depressed”[All Fields] OR “depression”[MeSH Terms] OR “depression”[All Fields] OR “depressions”[All Fields] OR “depressions”[All Fields] OR “depressive disorder”[MeSH Terms] OR (“depressive”[All Fields] AND “disorder”[All Fields]) OR “depressive disorder”[All Fields] OR “depressivity”[All Fields] OR “depressive”[All Fields] OR “depressively”[All Fields] OR “depressiveness”[All Fields] OR “depressives”[All Fields]).

### 2.2 Inclusion criteria

Inclusion criteria for this study: (1) different experimental rats and mice including C57BL/6J mice (213 cases), Wistar (110 cases) and SD rats (48 cases), (2) different depression models including chronic unpredictable mild stress (CUMS), maternal separation (MS), restraint stress, social defeat (SD) and Stop VRW, (3) behavior test including the forced swimming test (FST), sucrose preference test (SPT), and elevated plus maze (EPM) (Tables 1–3).

**Table 1:** Article basic information.

**References**	**Quantity (VRW and control)**	**Age**	**Sex**	**Species**	**Model**	**VRW time**	**Running distance (km/day)**	**Behavior test**
Dong et al. (2020)	15/15	4w	M	C57BL/6J	CUMS	21 month	/	(1)(2)(3)
Huang et al. (2017)	20/20	8w	M	C57BL/6J	CUMS	32 day	/	(9)(10)
Masrour et al. (2018)	8/8	3w	/	Wistar	Maternal separation	1 h (3 times a week)	/	(2)(5)(9)
Fuentes et al. (2021)	14/14	12w	M	C57BL/6	Maternal separation	20 day	6.81 ± 1.01	(1)(2)
Sadeghi et al. (2016)	12/12	8w	/	Wistar	Maternal separation	4 week	/	(1)(2)(3)(8)
Sahafi et al. (2018)	10/10	30d	/	Wistar	Maternal separation	15 week	/	(1)(3)
Marais et al. (2009)	12/12	8w	M	SD	Maternal separation and Chronic stress	4 week	/	(1)(8)
Maniam and Morris (2010)	12/12	2d	/	SD	Maternal separation and Chronic stress	6 week	0.24 ± 0.02	(1)
Lapmanee et al. (2013)	10/10	2d	M	Wistar	Restraint stress	6 week	11.08 ± 2.13	(1)(3)
Lapmanee et al. (2017)	8/10	2d	M	Wistar	Restraint stress	32 day	13.08 ± 2.92	(1)(2)(4)(5)
Ghalandari-Shamami et al. (2019)	6/6	2d	M	Wistar	Restraint stress	32 day	/	(1)(2)(4)(5)
Calpe-López et al. (2022)	20/12	2d	M	C57BL/6	Social defeat	6 week	/	(3)(4)
Pagliusi et al. (2020)	15/15	1d	M	C57BL/6	Social defeat	4 week	3.80 ± 0.72	(3)(4)
Morgan et al. (2019)	16/12	6w	F/M	C57BL/6	Stop VRW	6 week (2 h each day)	8.22 ± 1.00	(1)(4)(7)
Nishijima et al. (2013)	10/15	8w	M	C57BL/6	Stop VRW	6 week (2 h each day)	0.20 ± 0.02	(1)(4)(7)

(1) Forced swimming test. (2) Open field test. (3) Elevated plus-maze test. (4) Sucrose preference test. (5) Splash test. (6) Elevated zero maze. (7) Tail suspension test. (8) Elevated T-maze test. (9) Social interaction test. (10) Chemical nociceptive test.

**Table 2:** Maternal separation protocols in the selected studies.

**References**	**Protocol**
Marais et al. (2009)	Separated from mother for 180 min on the second day of life for 12 days and restraint stress on adult
Maniam and Morris (2010)	Separated from mother for 180 min or 15min on the second day of life for 12 days
Sahafi et al. (2018)	Separated from mother for 180 min on the second day of life for 13 days
Sadeghi et al. (2016)	Separated from mother for 180 min on the second day of life for 13 days
Maniam and Morris (2010)	Separated from mother for 180 min or 15 min on the second day of life for 12 days
Fuentes et al. (2021)	Separated from mother for 180 min on the first day of life for 21 days
Maniam and Morris (2010)	Separated from mother for 180 min on the second day of life for 22 days

**Table 3:** Other models protocols in the selected studies.

**References**	**Intervene method**	**Protocol**
Nishijima et al. (2013)	Stop VRW	VRW was stopped after 8 weeks of exercise, and the other rats continued to exercise until 21 weeks
Morgan et al. (2019)	Stop VRW	VRW was stopped after 4 months of exercise, and the other rats continued to exercise until 6 months
Pagliusi et al. (2020)	Social defeat	Swiss mouse varieties are attacked for 10 min each time for 10 days
Calpe-López et al. (2022)	Social defeat	OF1 mouse are attacked for 10 min for 4 each days
Kingston et al. (2018)	Social defeat	Resident mouse are attacked for 5 mins for 3 times
Lapmanee et al. (2017)	Restraint stress	Restraint stress for 4 weeks, 2 h a day
Ghalandari-Shamami et al. (2019)	Restraint stress	Restraint stress for 10 weeks, 2 h a day
Lapmanee et al. (2013)	Restraint stress	Restraint stress for 10 weeks, 2 h a day

### 2.3 Exclusion criteria

(1) Studies that were not randomized controlled trials (RCTs), (2) incomplete data, (3) not obtain full-text data were excluded. (4) No behavioral test index (5) an unclear number of rodents, (6) no modeling process were also excluded.

### 2.4 Data extraction

After removing duplicates, studies that did not meet the inclusion criteria for language or design were excluded. Two researchers independently screened the resulting literature and extracted data according to the inclusion and exclusion criteria. The extracted data were cross-checked and decisions were made by two researchers. Any disagreements were resolved through discussions with a third researcher. The abstract includes the author, year, age of the participants, duration of the experiment, sample size, intervention measures, intervention duration, and outcome indicators (Naghshvarian et al., 2017).

### 2.5 Quality evaluation

Two researchers independently assessed the quality of the literature on 10 aspects using the SYRCLE Animal Experiment Risk Assessment Tool manual. If there were any disagreements, a third researcher was invited to make a judgment. To assess publication bias, we used a modified version of the SYRCLE Risk-of-Bias tool (Hooijmans et al., 2014). We assigned ratings of “+”, “–,” or “?” for each domain of the instrument. If the reported details were insufficient, we could assign internal and external validity scores to the studies included in the review. No studies were excluded based on quality evaluations.

## 3 Results

### 3.1 Literature search and screening

**Figure 1:**
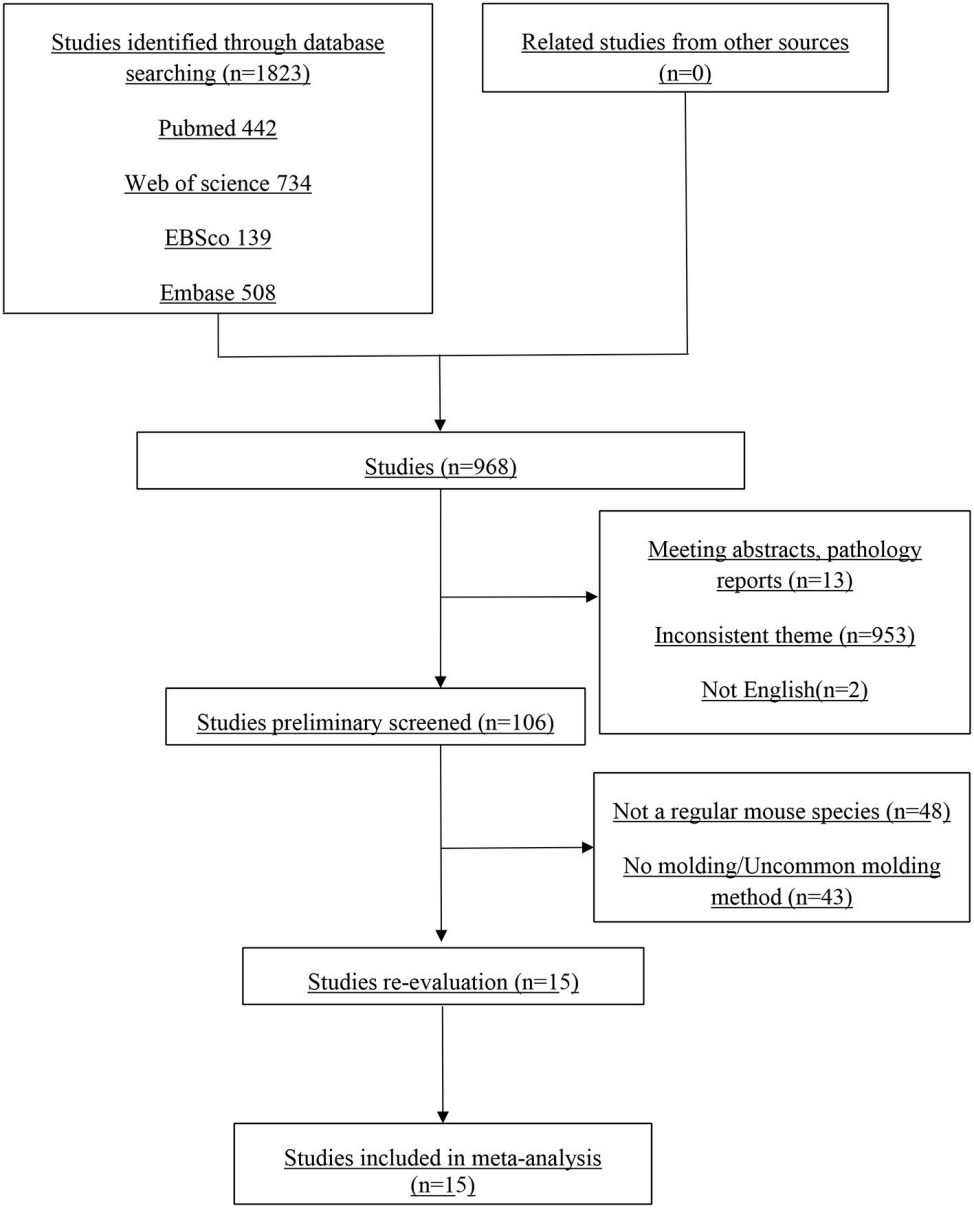
Flowchart of study selection.

As shown in Figure 1, the study selection process involved a total of 1,832 articles initially. After removing duplicates, 968 papers remained. Subsequently, 15 articles were screened based on their titles and abstracts. After this screening process, the study design was reevaluated, and 15 full-text studies were deemed suitable for inclusion in this review and were considered in the meta-analysis.

### 3.2 Assessment of risk bias of included studies

The 15 studies included in the analysis were assessed using the SYRCLE bias risk tool, and 10 investigations were deemed suitable for inclusion. The evaluation resulted in a total score of 10 points, with each study receiving one point for low risk, as detailed in Table 4. The criteria used for evaluation were as follows: whether the allocation sequence was sufficiently/correctly generated or applied; whether the groups had the same baseline or adjusted for confounding factors; whether allocation hiding was sufficient/correct; whether the animals were randomly placed during the experiment; whether animal breeders and researchers were blinded to the interventions received by the animals; whether the animals in the outcome evaluation were randomly selected; whether the outcome assessors were blinded; whether incomplete data were adequately/correctly reported; whether the study report was related to selective outcome report; and whether there were no other issues that created a high risk of bias. The symbol “–” represents low risk; “+” represents high risk; and “?” represents uncertainty.

**Table 4:** SYRCLE bias risk.

**References**	①	②	③	④	⑤	⑥	⑦	⑧	⑨	⑩	**Score**
Dong et al. (2020)	?	?	?	?	?	?	+	?	?	+	2
Huang et al. (2017)	?	?	?	?	?	?	+	?	?	+	2
Masrour et al. (2018)	?	?	?	?	?	?	?	?	?	+	1
Fuentes et al. (2021)	?	?	?	+	?	?	?	?	?	+	2
Sadeghi et al. (2016)	?	?	?	+	?	?	?	?	?	+	2
Sahafi et al. (2018)	–	?	?	+	?	?	?	?	?	+	1
Marais et al. (2009)	–	?	?	+	?	?	?	?	?	+	1
Maniam and Morris (2010)	?	?	?	+	?	?	+	?	?	+	3
Lapmanee et al. (2013)	?	?	?	+	?	?	+	?	?	+	3
Lapmanee et al. (2017)	–	?	?	?	+	?	+	?	?	+	2
Ghalandari-Shamami et al. (2019)	?	?	?	+	?	?	+	?	?	+	3
Calpe-López et al. (2022)	?	?	?	+	?	?	+	?	–	+	3
Pagliusi et al. (2020)	?	?	?	+	?	?	+	?	?	+	3
Morgan et al. (2019)	–	?	?	+	?	?	?	?	?	+	1
Nishijima et al. (2013)	?	?	?	+	?	?	?	?	?	+	2

### 3.3 Meta-analysis

#### 3.3.1 EPM

**Figure 2:**
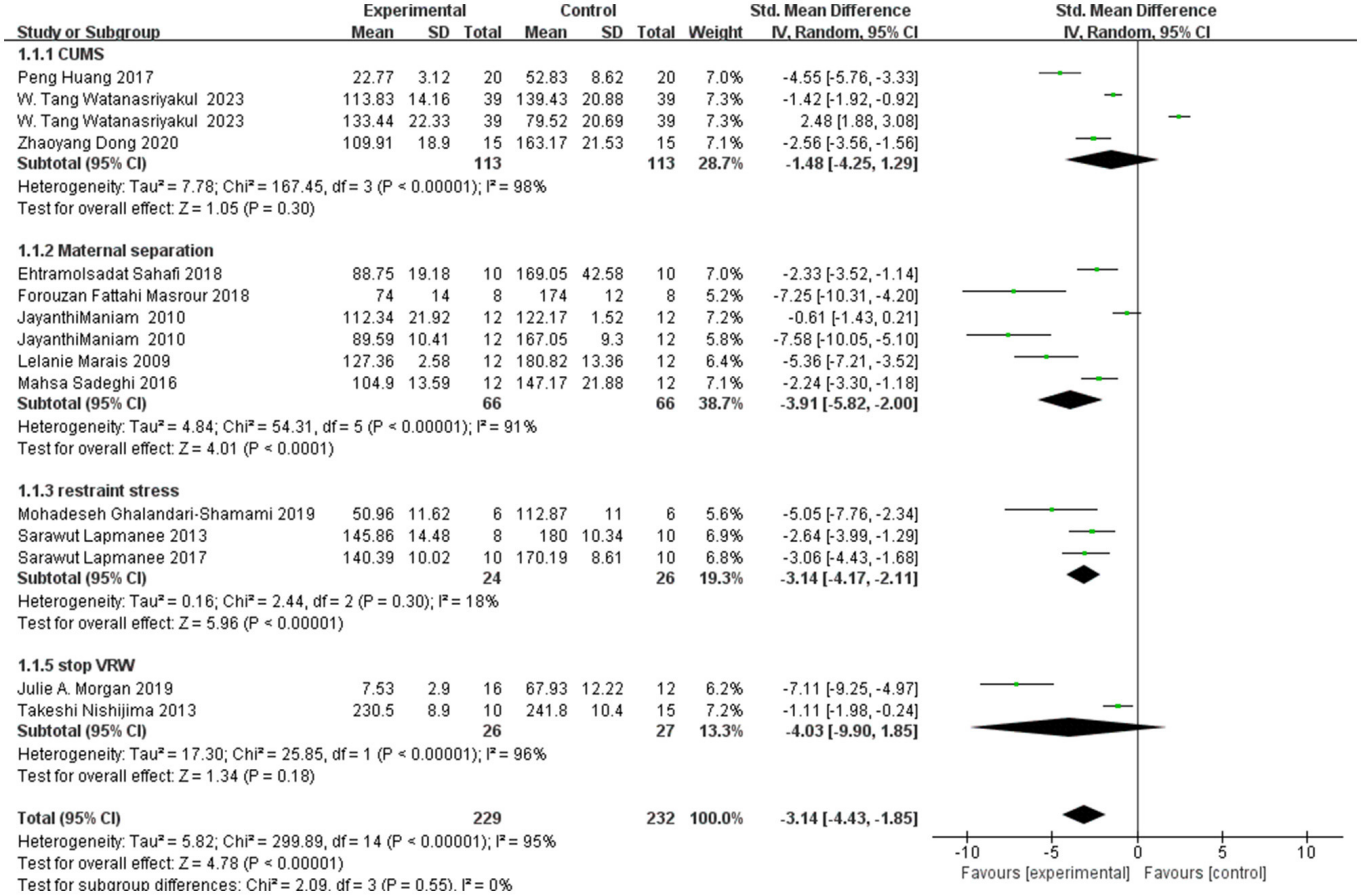
Time in open arm in the EPM.

There was 97% heterogeneity in the MS group, 88% heterogeneity in the Restraint Stress group, 99% heterogeneity in the social defeat group, and no heterogeneity in the CUMS group or STOP VRW due to an insufficient number of articles. The final overall heterogeneity was 96%, and the results were significantly different [SMD = 0.83, 95% CI (0.47, 1.19)]. The confidence interval does not contain 0, indicating that there is a significant difference in effect between the two mean groups. Further details are shown in Figure 2.

#### 3.3.2 FST

**Figure 3:**
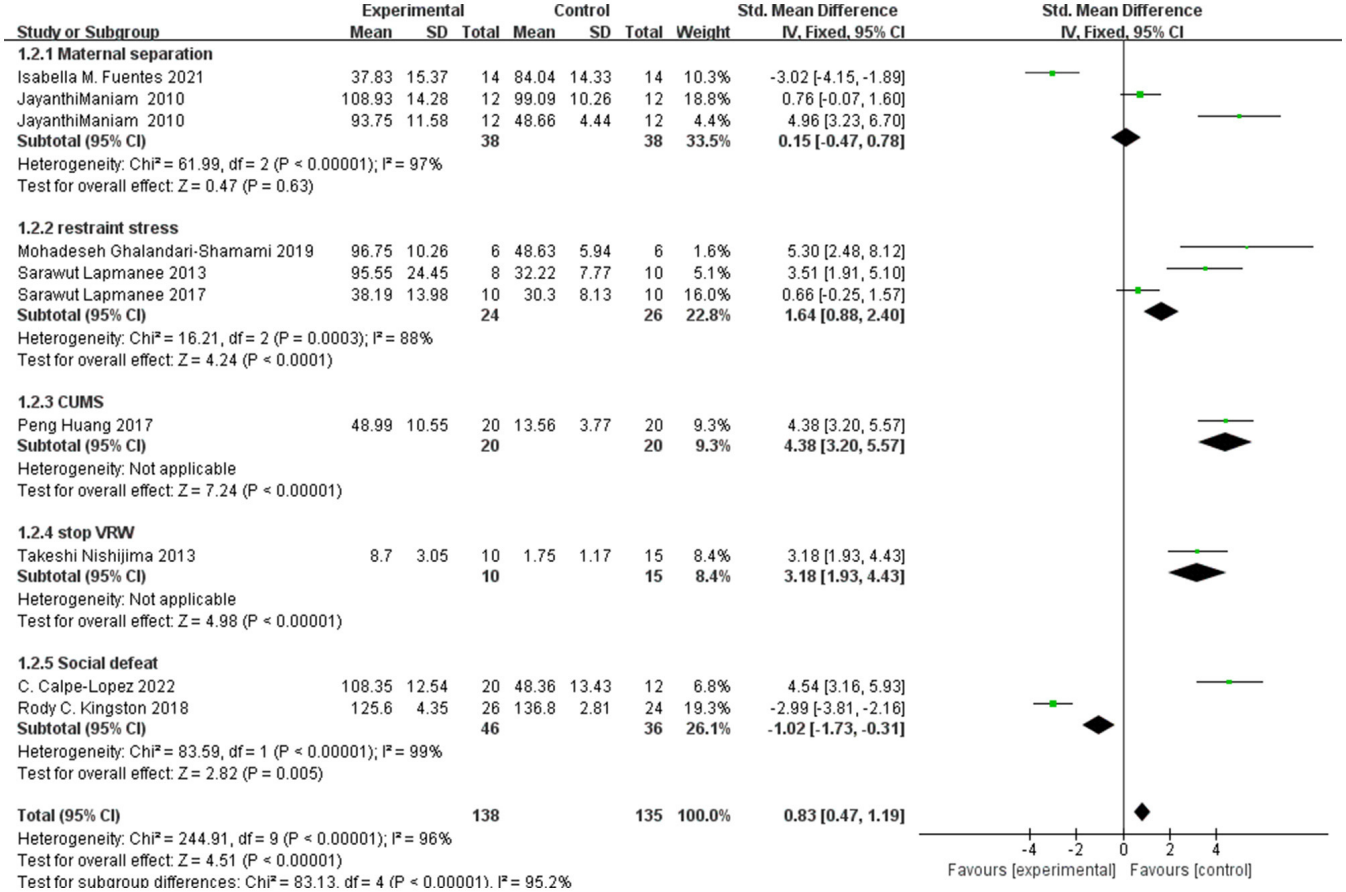
Immobility index in FST.

The overall heterogeneity between the study groups was *I*^2^ = 98%, which was >50%. Heterogeneity of 70% or higher indicates significant heterogeneity, making the results questionable. Heterogeneity of more than 70% means that researchers should consider not performing a meta-analysis of the results, as high heterogeneity may indicate that the effects shown in the meta-analysis are not valid. However, the heterogeneity of restraint stress was only 18% in the unpredictable mild stress stimulus (CUMS) group, 91% in the MS group, and 96% in the STOP-VRW group. The difference in the restraint stress immobility index after the exercise intervention was statistically significant [SMD = −3.14, CI (−4.17, −2.11)]. The confidence interval does not contain 0, indicating that there is a difference effect between the two groups of mean. The larger the area of the diamond square, the greater the weight in the analysis. Further details are shown in Figure 3.

#### 3.3.3 SPT

**Figure 4:**
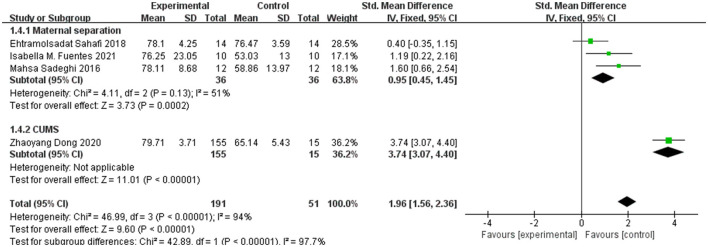
Sucrose consumption index.

Four articles tested the sucrose consumption index in SPT, with a 51% heterogeneity between MS groups. However, there were not enough articles to analyze the sucrose consumption index in CUMS. The data was analyzed using a random effects model. The sucrose consumption index was found to be higher in the observation group than in the control group [SMD = 1.96, 95% CI (1.56, 2.36)]. The confidence interval does not contain 0, indicating a significant difference between the mean values of the two groups. More details can be found in Figure 4.

## 4 Discussion

The systematic review and meta-analysis we have described highlights both the potential benefits of voluntary wheel running exercise for depression treatment across various modeling methods, as well as the challenges in interpreting the results due to high heterogeneity between studies. The finding that voluntary wheel running exercise had a significant effect on depression treatment, regardless of the modeling method used, is encouraging. It suggests that this non-pharmacological intervention may have broad applicability in addressing depression caused by different stressors and scenarios. However, the high heterogeneity between articles is a significant limitation that needs to be acknowledged. Heterogeneity in meta-analysis can arise from a variety of sources, including differences in study design, participant characteristics, intervention protocols, and outcome measures. This variability can make it difficult to draw definitive conclusions from the data and can reduce the accuracy and reliability of the results. To address this issue, future research should aim to standardize study protocols and outcome measures as much as possible. This would help reduce the heterogeneity between studies and improve the overall quality of the evidence base. Additionally, researchers should carefully consider the potential sources of heterogeneity in their meta-analysis and use statistical methods to account for it, such as sensitivity analyses or subgroup analyses.

Despite the limitations, the systematic review and meta-analysis we've described provides valuable insights into the potential role of voluntary wheel running exercise in depression treatment. The results suggest that this intervention may hold promise as an adjunctive therapy for depression, and further research is needed to explore its effectiveness and mechanisms of action.

### 4.1 Model characteristics and behavioral test

It is well-established that rodents cannot fill out questionnaires or scales to directly express their emotional states such as anxiety or helplessness, making the assessment of depression reliant on external behavioral analysis (Kroenke et al., 2009; Wang and Gorenstein, 2013), While behavioral tests provide a simple and efficient method of evaluating these emotional states, they do not always accurately reflect the degree of depression due to the potential for significant errors in interpretation (Harro, 2019). For instance, the forced swim test records of immobility, during which the animal initially tries to escape but eventually becomes immobile. This immobility may reflect a certain level of behavioral desperation. By observing and recording the immobility time of an animal in a state of desperation, we can understand the animal's response to external stress and assess the level of depression. We compared 10 studies to determine the immobility index during the forced swim test, which is an environment where the animal desperately tries to escape but is unable to do so, creating an inescapable stressful situation. After a certain period of time, the animal enters a typical “immobility state,” which can be used to evaluate the effects of antidepressants. The increased immobility time in rats during the forced swim test (FST) has been interpreted as a sign of despair, but it could also be a sign of adaptation to the water environment (Yankelevitch-Yahav et al., 2015). Several articles consistently report a duration of 5 min (Sahafi et al., 2018; Ghalandari-Shamami et al., 2019) immediately following the placement of water, whereas contrasting sources specify a slightly longer period of 6 min (Huang et al., 2017; Dong et al., 2020). Based on our personal testing result, it is evident that the stationary phase commences later in the timeline. Consequently, when converting the data, the stationary duration recorded in the articles indicating 6 min will exceed that of the 5-min timeframe. Similarly, the chronic unpredictable mild stress (CUMS) model, which involves exposing rats to various stressors, has been criticized for its lack of standardization and potential to yield abnormal results in the FST due to factors like cold and hot swimming. For example, Tatyana Strekalova suggests using the sucrose preference test (SPT) as a reasonable behavioral test method to evaluate anhedonia caused by CUMS (Strekalova et al., 2022). Additionally, classifying CUMS mice into “resilient” and “susceptible” cohorts can help provide more reasonable interpretations of behavioral test results (Antoniuk et al., 2019). The modeling process of CUMS varies widely among studies, making it difficult to widely promote the model without a unified protocol. Efforts have been made to standardize the modeling process and connect behavioral test results with depression models through reasonable interpretation. The open-field test and elevated plus-maze test have been found to be effective for evaluating the CUMS model, and it is recommended that other researchers also conduct this type of study (Hu et al., 2017). Andrea Raez offers a novel approach to analyzing behavioral tests by combining the FST with helplessness reactions to analyze their relationship (Ráez et al., 2020). In summary, while behavioral tests are valuable tools for evaluating emotional states in rats and mice, their interpretation must be approached with caution due to the potential for errors and variability. Future research should focus on standardizing modeling protocols, developing new behavioral tests, and refining interpretation methods to improve the accuracy and reliability of depression models in rodents.

### 4.2 Voluntary wheel-running exercise

In general, exercise has been shown to exert profound effects on the nervous system, contributing to the alleviation of depression (Cotman and Berchtold, 2002; Ma, 2008). Voluntary exercise, a subject of increasing interest in recent years, has not been as extensively studied as forced exercise, though it has been explored in terms of factors such as BDNF (Bastioli et al., 2022), tyrosine hydroxylase-positive neuron (Tsai et al., 2019) and plasma kynurenine (KYN) level (Su et al., 2020) Voluntary wheel running, a more natural form of exercise, offers the distinct advantage of providing more effective rewarding feedback (Mul, 2018; Stranahan et al., 2008), which is associated with activation of both the sympathetic nervous system, leading to epinephrine production, and the hypothalamic-pituitary-adrenal (HPA) axis. However, the precise mechanisms underlying these effects remain elusive (Richter et al., 2014). In comparison to forced exercise, voluntary wheel running is characterized by significantly faster speeds, with some mice running several times faster over the same distance (Leasure and Jones, 2008). With regards to movement distance, the study revealed considerable variability among mice, both within and between strain. Body size contributed to the differences in movement distances between large and small mice, yet significant variations were also observed within the same mouse strain. For instance, while the least active C57BL/6 mice moved an average of only 0.2 km/day, the most active ones traveled as much as 6.8 km/day. Interestingly, all mice in the study exhibited an increase in their weekly movement distance, with the exception of one SD rat that not only moved minimally (0.24 km/day) but also showed a decreasing trend in movement distance. This particular rat was suspected to be high-fat-fed, and its declining willingness to engage in voluntary movement was likely due to obesity-induced changes in the brain that reduced its motivation to move (Maniam and Morris, 2010). Overall, the findings suggest that free running on a treadmill may be beneficial in ameliorating symptoms of depression or anxiety. However, whether the length of the running distance has a definitive impact on depression remains an open question that necessitates further experimental investigation.

## Data Availability

The raw data supporting the conclusions of this article will be made available by the authors, without undue reservation.
